# In Reply: Body Mass Index and the Risk of Poor Outcome in Surgically Treated Patients With Good-Grade Aneurysmal Subarachnoid Hemorrhage

**DOI:** 10.1227/neu.0000000000002058

**Published:** 2022-06-10

**Authors:** Ilari Rautalin, Seppo Juvela, R. Loch Macdonald, Miikka Korja

**Affiliations:** * Department of Neurosurgery, University of Helsinki and Helsinki University Hospital, Helsinki, Finland; ‡ Community Health Partners, Community Neurosciences Institute, Fresno, California, USA

To the Editor:

We want to thank Drs Wang and Yang for their great interest and feedback regarding our recent article in *Neurosurgery*.^1^ In their Letter,^2^ Drs Wang and Yang have pointed out 4 factors, namely (1) age, (2) cohort heterogeneity, (3) aneurysm size, and (4) preoperative condition, that may have affected our presented results. In short, Drs Wang and Yang raise concerns as to whether these factors are outcome predictors in our study and, therefore, explain at least partially the reported association between high body mass index (BMI) and poor aneurysmal subarachnoid hemorrhage (aSAH) outcome. Below, we clarify the study concept in more detail in light of these factors.

First, the authors have correctly noted that the age of patients with aSAH with poor outcomes (Glasgow Outcome Score 1-3) was higher than that of the patients with favorable outcome (Glasgow Outcome Score 4-5). However, we treated age in our analyses with multiple methods, which showed that increasing age did not significantly affect the association between BMI and poor outcome. Specifically, we reported that the median BMI did not differ between young (<50 years) and old (≥50 years) patients with aSAH (Table 1). In addition, we adjusted the risk estimates of BMI for increasing age in both partially and fully adjusted models (Figure 2). Finally, we assessed the effect of BMI on aSAH outcome in subgroups of young and old patients with aSAH (Table 2). Based on these analyses, our results strongly suggest that although older age increases the risk of poor outcome after aSAH in general, it does not confound the association between BMI and aSAH outcome identified in our comprehensive analyses.

Second, we agree with the authors that the inclusion of patients with aSAH from multiple centers, countries, and decades has likely resulted in a vast study cohort heterogeneity. Moreover, it is also possible that some of the patients with aSAH who underwent surgical treatment 20 or 30 years ago would have undergone endovascular treatment nowadays. However, contrary to the authors' opinion, the multicenter and long-term design in fact increases—not decreases—the external validity of our findings. This fact is further supported by the observation that the adverse effect of obesity was evident in all included study cohorts, with no substantial between-cohort heterogeneity (Figure 1). Furthermore, as underlined several times in the article, our study focused on surgically treated patients with aSAH and, therefore, future studies are needed to investigate whether the findings are applicable to endovascularly treated patients.

Third, Drs Wang and Yang state that patients with aSAH with aneurysms more than 20 mm in diameter have an increased risk of poor outcome and this could somehow distort our findings. Evidence to support this is scarce as high-quality studies that associate BMI with the size of aneurysms are lacking. Hence, it is somewhat challenging to speculate how this would affect our findings and conclusions. Unfortunately, our pooled data set did not include data on the size of aneurysms. However, even if size more than 20 mm is a confounder in our BMI-related analyses (ie, the size associates with both BMI and poor outcome), the number of such large aneurysms is very low, especially among patients with good-grade aSAH (our cohort), and, therefore, it is highly unlikely that the results and conclusions are skewed.

Fourth, there may be some misunderstanding regarding the authors' last concern about our limited consideration of the preoperative condition of patients with aSAH as the strictly defined preoperative condition was in fact one of the main cornerstones of our article. As written in the article, we only included patients with good-grade aSAH (defined as patients with Glasgow Coma Scale 13-15 or World Federation of Neurological Surgeons grades I-III). Moreover, we also reported the extent of bleeding [based on the (modified) Fisher Scale as presented in the Supplementary File] in Table 1. Although patients with aSAH with thick bleedings suffered more commonly from poor outcome, we found no difference in BMI between the patients with no/thin and thick bleedings. Thus, the extent of bleeding was not considered as a confounder in our BMI-related analyses.

To summarize, we thank Drs Wang and Yang for highlighting several factors that may have raised concerns with our findings and interpretations. Based on the previous literature, our comprehensive data analyses and the given clarifications, we believe that the reported positive association between high BMI and poor outcome after aSAH is scientifically sound finding and relevant for the readers of *Neurosurgery*.
